# Hypermethylated *GRIA4*, a potential biomarker for an early non-invasive detection of metastasis of clinically known colorectal cancer

**DOI:** 10.3389/fonc.2023.1205791

**Published:** 2023-07-05

**Authors:** Eva Lukacova, Tatiana Burjanivova, Petar Podlesniy, Marian Grendar, Eva Turyova, Ivana Kasubova, Ludovit Laca, Peter Mikolajcik, Eva Kudelova, Andrea Vanochova, Juraj Miklusica, Sandra Mersakova, Zora Lasabova

**Affiliations:** ^1^ Department of Molecular Biology and Genomics, Comenius University in Bratislava, Jessenius Faculty of Medicine in Martin (JFM CU), Martin, Slovakia; ^2^ Centro Investigacion Biomedica en Red Enfermedades Neurodegenerativas (CiberNed), Madrid, Spain; ^3^ Laboratory of Bioinformatics and Biostatistics, Biomedical Center Martin JFM CU, Commenius University in Bratislava, Jessenius Faculty of Medicine in Martin (JFM CU), Martin, Slovakia; ^4^ Biomedical Center Martin, Jessenius Faculty of Medicine in Martin, Comenius University in Bratislava, Martin, Slovakia; ^5^ Clinic of Surgery and Transplant Center, Jessenius Faculty of Medicine in Martin, Comenius University in Bratislava, Martin, Slovakia

**Keywords:** colorectal cancer, methylation, ctDNA, metastasis, liquid biopsy

## Abstract

**Introduction:**

Colorectal cancer (CRC) can develop through several dysregulated molecular pathways, including the serrated pathway, characterized by CpG island methylator (CIMP) phenotype. Although the tumor tissue is a commonly tested material, sample types such as stool or plasma, bring a new, non-invasive approach. Several cancer-related methylated genes have been identified in CRC patients, including gene *GRIA4*, showing promising diagnostic potential. The aim of our study was to develop a sensitive droplet digital PCR (ddPCR) assay to examine *GRIA4* hypermethylation status in CRC patients and evaluate its diagnostic potential in tissue and liquid biopsy samples.

**Methods:**

In total, 23 patients participated in this study, 7 patients with primary CRC and 16 patients with liver metastasis of clinically known CRC. We obtained tumor and non-tumor tissues (N=17), blood samples pre- and post-surgery (N=22), and blood of five volunteers without a personal cancer history. We have developed and optimized a ddPCR assay for *GRIA4* hypermethylation detection, from tissue and plasma samples.

**Results:**

We detected significantly increased *GRIA4* methylation in tumor tissues compared to their adjacent non-tumor tissue, p<0.0001. Receiver operating characteristic (ROC) analysis defined cutoff values to separate primary tumors and metastases from non-tumor colon/rectum, specifically 36.85% for primary tumors and 34.81% for metastases. All primary tumors were above this threshold. When comparing the methylation levels of metastatic vs. non-tumor tissue, a smaller increase was observed in liver metastasis versus colon tissue (3.6× gain; p=0.001), then in liver metastasis versus adjacent liver tissue (17.4× gain; p<0.0001). On average, *GRIA4* hypermethylation in primary tumor plasma was 2.8-fold higher (p=0.39), and in metastatic plasma, 16.4-fold higher (p=0.0011) compared to healthy individuals. Hypermethylation in metastatic plasma was on average 5.9 times higher (p=0.051) than in primary tumor plasma. After tumor removal surgery, average hypermethylation decrease in plasma was 1.6× for primary (p=0.037) and 4.5× for metastatic patients (p=0.023).

**Discussion:**

Based on our data, it can be inferred that *GRIA4* serves as a tissue specific biomarker for the colon/rectum tissue, thus is suitable for cancer classification. This biomarker showed the potential to be an attractive target for early non-invasive detection of metastases of clinically known CRC, although additional analysis has to be performed.

## 1 Introduction

Colorectal cancer (CRC) is the third most prevalent cancer in men, after lung and prostate cancer, and the second most common cancer in women, after breast cancer (1). According to Globocan 2020 (2), 4,821 new cases of colorectal cancer were diagnosed in 2020 in Slovakia, which makes it the most common form of cancer in the Slovak Republic. The most common site of distant metastasis for colorectal cancer is the liver, due to the direct connection through the portal vein (3). Approximately 50% of patients with colorectal cancer develop liver metastases during the course of their disease (4).

The development of CRC can proceed through the accumulation of genetic and epigenetic changes (5). DNA methylation is the most common epigenetic modification. It ensures cell-specific gene expression for normal development, cell functioning, and tissue stability. On the other hand, in somatic cells, hypermethylation/hypomethylation within the specific promoter region can contribute to neoplastic cell transformation (6). In 1999, Toyota et al. (7) proposed the term CIMP (CpG island methylator phenotype) to describe a subset of CRCs that show extensive hypermethylation of CpG dinucleotides.

Multiple sample types can be used for the identification of epigenetic alterations in CRC patients. Although tumor tissue is the predominant choice, non-invasive approaches, such as stool or plasma sampling, are progressively being incorporated into clinical practice. Circulating tumor DNA (ctDNA) is tumor-derived DNA, present in body fluids, such as blood, stool, urine, and saliva (8, 9). It is released into the bloodstream by tumor cells undergoing apoptosis/necrosis or *via* active secretion (10). Due to its origin, ctDNA provides comprehensive genetic and epigenetic information about tumor, and its concentration can greatly vary, depending on tumor size and type, proliferative stage, response to the treatment, level of vascularization, etc. (11). Circulating tumor DNA can be used to monitor disease dynamics non-invasively. It has the potential to assess therapy response and efficacy (12–14) and predict and improve early relapse detection (15, 16).

A large number of cancer-related methylated genes have been identified in CRC patients, for instance, *MLH1*, *CDKN2A*, *MGMT* (17, 18), *SFRP2* (19), *Vimentin* (20), *BMP3* (21), *Sept9* (22), *NDRG4* (23), and many others, in the last few decades. Currently, a limited number of assays for non-invasive hypermethylation detection are commercially available, for example, assays for the detection of methylated *Sept9* (Epi proColon^®^) from plasma (24), *Vimentin* (ColoSure™) (25), and *BMP3* together with *NDRG4* (Cologuard^®^) from fecal DNA (26). When searching for other epigenetic biomarkers, gene *GRIA4* (glutamate ionotropic receptor AMPA subunit 4) has great diagnostic potential in patients with colorectal cancer, although studies on its biological properties are quite limited. It was published recently that 100% tissue and 71.3% plasma samples of metastatic CRC patients had a higher methylation profile for *GRIA4* gene (27). In another study, gene *GRIA4* showed hypermethylation in 99.1% of experimental tissue samples (28). The presence of methylated *GRIA4* promoter was also observed in stool specimens, indicating its potential utility as a biomarker for the early detection of colorectal cancer from stool samples (29). The investigations conducted by and Sun et al. (30) and Hauptman et al. (28), both in 2019, employed the TCGA dataset to examine CRC methylation biomarkers, including hypermethylated *GRIA4*. Sun et al. aimed to validate a previously identified markers, while Hauptman et al. sought to identify potential CRC biomarkers from TCGA data. Among 198 genes, *GRIA4* exhibited the most significant methylation difference among six selected genes. All four promoter probes of the *GRIA4* gene displayed high methylation difference, with two of them present in 98.4% of the samples. Furthermore, *GRIA4* was found to be downregulated in 98.1% of the samples within the TCGA dataset.

Circulating DNA can be extracted from plasma and identified using a variety of molecular techniques. Analyzing tumor material acquired by liquid biopsies necessitates very sensitive assays. Multiple ctDNA analysis platforms are currently available, but PCR-based techniques are still the backbone of all detection strategies. Droplet digital PCR (ddPCR) is a sensitive, low-cost detection method that has been commercially available since 2011 (31). This technique is suitable for targeting specific mutations/methylations/alterations on DNA fragments present at very low concentrations. However, the complexity of laboratory protocols, the constraint in the number of targets being tested, and the variability in analytical sensitivity could potentially impose limitations on the application of this technique (32). The aim of our study was to develop sensitive droplet digital assay to examine *GRIA4* methylation status in CRC patients, either with a primary tumor or with metastases. The next step was to evaluate its diagnostic potential using non-invasive liquid biopsy samples.

## 2 Patients and methods

### 2.1 Patients

In total, 23 patients participated in this study (**Supplementary Table S1**), 7 patients with primary tumor (**Supplementary Table S2**) and 16 patients with liver metastasis of clinically known colorectal cancer. Tumor tissues of 17 CRC patients (5 primary and 12 metastatic) and blood samples of 22 CRC patients (6 primary and 16 metastatic) and of 5 volunteers without a personal cancer history were obtained in collaboration with Clinic of General, Visceral and Transplantation Surgery and Department of Pathology at the Jessenius Faculty of Medicine (Comenius University), University Hospital in Martin. Blood samples of patients were taken before and after surgical removal of the tumor. The histopathological diagnosis was conducted by the experienced pathologists. Histological typing, grading, localization, and staging of tumors were determined using the recommendation according to WHO and Union for International Cancer Control (UICC) (33). This study was approved by the Ethics Review Board of the Jessenius Faculty of Medicine.

### 2.2 DNA isolation and quantification, bisulfite conversion

Tumor and non-tumor samples were obtained after resection surgery with subsequent evaluation of the tissue by an experienced pathologist. Genomic DNA from tumor tissues was isolated with the commercial kit DNeasy Blood and Tissue (Qiagen, Hilden, Germany) according to manufacturer’s instructions. Isolated DNA was eluted into 60 μl and stored at −20°C. EpiTect Bisulfite Kit (Qiagen, Hilden, Germany) was used for bisulfite conversion of gDNA. Converted DNA was eluted into 30 μl and stored at −20°C. Blood samples were collected to ethylenediaminetetraacetic acid (EDTA) tubes and centrifuged at 2,200*g* for 8 min at 4°C. Plasma was pipetted into new 1.5-ml tubes and centrifuged one more time at 20,000*g* for 8 min at 4°C. Plasma samples were then stored at −80°C until cfDNA extraction was performed. Circulating DNA extraction from plasma samples and bisulfite conversion were performed according to the manufacturer’s instructions of Epi proColon 2.0 CE Plasma Quick Kit (Epigenomics AG, Berlin, Germany) from 3.5 ml of plasma. Bisulfite-converted DNA was then eluted into 60 μl, stored at 4°C, and used for analysis within 24 h. The rest of the eluate was stored at −20°C. For DNA quantification, Qubit dsDNA HS assay kit (Life Technologies, CA, USA) and Qubit 2.0 fluorometer were used.

### 2.3 Droplet digital PCR

Droplet digital PCR was performed in 20 μl ddPCR reactions, containing 10 μl Supermix for Probes (No dUTP) (Bio-Rad Laboratories, Hercules, CA, USA), 1.4 μl primers (final concentration, 225 nM) and probes (final concentration 125 nM) (**Table 1**), and 8.6 μl of circulating DNA or 0.6 μl of genomic DNA adjusted with water up to volume 8.6 μl. The probe complementary to the methylated sequence of the *GRIA4* promoter is referred as M-Probe, and the one complementary to the unmethylated sequence is referred as U-Probe (**Table 1**). As controls, commercially available, fully methylated and fully unmethylated, EpiTect DNA controls (Qiagen, Hilden, Germany) and ultrapure water were used to check for template contamination. The reaction (20 μl) from the previous step was transferred to the middle rows of a DG8 (Bio-Rad Laboratories, Hercules, CA, USA) cartridge. After that, 70 μl of Droplet Generation Oil for Probes was loaded into the bottom wells of DG8. Cartridge was then placed into the QX200 droplet generator, which produces approximately 20,000 droplets per sample. Created droplet emulsion (40 μl) was then pipetted from the top wells of the cartridge into 96-well plate. The PCR plate was covered with pierceable foil and heat sealed using Bio-Rad’s PX1. It was placed in a T100 thermal cycler (Bio-Rad Laboratories, Hercules, CA, USA), and the protocol was initialized with denaturation (95°C, 10 min), following 40 cycles of denaturation (94°C, 30 s), annealing/extension (50°C–62°C during optimization, then 56°C, 1 min) and droplet stabilization (98°C, 10 min.) with ramps of 2°C/s. After PCR, the product was held at 4°C with cooling ramp set up ~1°C/s, until the next step of analysis.

**Table 1:** Primer and probe sequences for *GRIA4* gene.

Primer	Sequence		Tm °C
Forward	5′-CACCACAACCACCACACACA-3′		55.2
Reverse	5′-CCTTACTTTCTCACATACACACAA-3′		54.6
Probe	Sequence	Tm°C	54.6
U-Probe	5′-CACCACAACCACCACACACA-3′	61.0	54.6
M-Probe	5′-CGCCGCGACCGCCACAC-3′	67.2	54.6

U-Probe is complementary to the unmethylated sequence of the *GRIA4* promoter, M-Probe is complementary to the methylated sequence of the *GRIA4* promoter.

### 2.4 Droplet analysis using QX200™ droplet reader and data interpretation in QuantaSoft™ software

After amplification, a 96-well plate was loaded to the QX200 Droplet Reader (Bio-Rad Laboratories, Hercules, CA, USA), where droplet analysis of each well was carried out. Each droplet was analyzed using QuantaSoft software (Bio-Rad Laboratories, Hercules, CA, USA) and divided into four clusters according to fluorescence emission analysis in HEX or FAM wavelengths. Droplets containing methylated DNA with high FAM amplitude, droplets containing unmethylated DNA with high HEX amplitude, and droplets with both types of DNA with high HEX and FAM amplitudes and empty droplets without target DNA. Data obtained from QX200 Droplet Reader were analyzed and interpreted by QuantaSoft v.1.7 Software (Bio-Rad Laboratories, Hercules, CA, USA). The correlation coefficient (R²) was calculated from serial dilutions of 100% methylated EpiTect control DNA and 100% unmethylated control DNA into water with 8,000, 4,000, 2,000, 1,000, 500, 250, 125, and 62.5 copies/per reaction as a correlation between two variables, expected number of copies and detected number of copies. Selectivity of each probe was calculated from the analysis of diluted controls in a pair with the opposite probe, more precisely a methylated control with a U-probe and an unmethylated control with an M-probe. Detected copies were divided by number of copies detected for each control in pair with the complementary probe, more precisely a methylated control with an M-probe and an unmethylated control with a U-probe. Specificity was calculated for each dilution, and the final number was an average of all values determined. Threshold values were defined during assay development and optimization processes, 1,500 for FAM and 2,500 for HEX (genomic DNA) and 1,500 for FAM and 2,700 for HEX (circulating DNA). The quantity of methylation was expressed in percentage as the ratio of methylated sequences to the sum of methylated and unmethylated sequences.

### 2.5 Statistical analysis

Data were explored and analyzed in R, ver. 4.0.5, with the aid of different libraries (34–48). Data and R script to reproduce the presented results are available at Mendeley data repository (49). For exploratory data analysis, data were summarized by the mean, SD, min, quartiles, and max. Spaghetti plot was used to visualize the methylation values in pairs. The boxplot overlaid with swarmplot and quantile–quantile plot with the 95% confidence band constructed by bootstrap was used to assess normality of the data. For the regression model, linear mixed model (LMM) was used to implement repeated measures ANOVA with non-constant variance, i.e., to model the association between methylation and interaction of group and specimen. Non-homogeneity was taken into account using the weights in the lme() function of nlme library. For the specimen data, the methylation was log-transformed to obtain a fit passing diagnostic analysis. In addition, one subject with extremely high methylation in plasma was excluded from the data prior to the model fitting. Effect size was quantified by the marginal and conditional R^2^. Marginal means were estimated given the fitted model and the grid of the factors. Contrasts, based on the *a priori* research questions, were specified, and the resulting p-values were adjusted using the Benjamini–Hochberg correction. The interaction plot was used to visualize the marginal means and their 95% confidence intervals. For the LMM model of methylation in plasma, the marginal means were back-transformed. Methylation in plasma before surgery for primary tumor *vs*. healthy controls was compared by the two-sample t-test, after assessing normality of the data by the quantile–quantile plot with the 95% confidence band constructed by bootstrap and by boxplot overlaid with swarmplot. For the case of metastasis, the methylation in plasma before surgery appeared skewed to the right; hence, the data were log-transformed prior to performing the two-sample t-test. EDA suggested that the log-transformation was appropriate. The cutoff on methylation to best separate between tumor and normal (separately in primary; in metastases) was obtained as the cutoff corresponding to the Youden index on the empirical receiver operating characteristic (ROC) curve.

## 3 Results

### 3.1 Droplet digital PCR as specific and sensitive method for detection of methylated DNA in tissue and plasma sample

**Figure 1:**
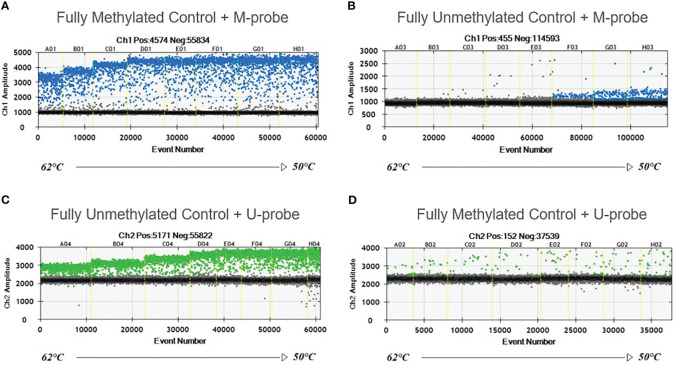
Temperature gradient for optimizing annealing temperature. Eight ddPCR reactions in temperature gradient ranging from 50°C to 62°C. Positive droplets at higher amplitude (blue or green) are methylated **(A, B)** or unmethylated **(C, D)**. Negative droplets (gray) at low amplitude are without the amplification. Unmethylated control with M-probe **(B)** and methylated control with U-probe **(D)** were used to detect unspecific binding.

**Figure 2:**
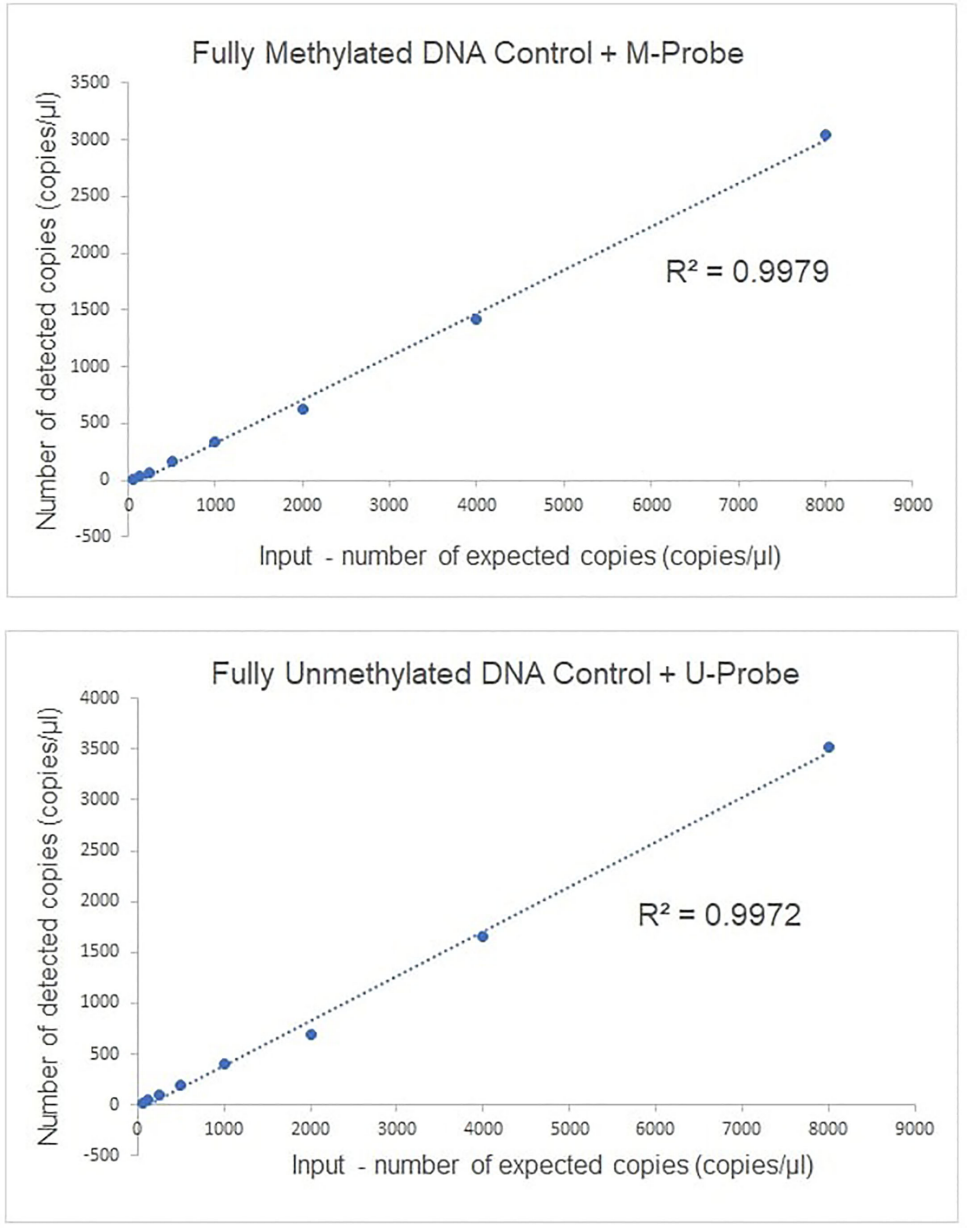
The linearity of ddPCR assays for methylated *GRIA4* detection and quantification. Linearity of ddPCR assay is shown using serially diluted EpiTech control DNAs (from 8,000, 4,000, 2,000, 1,000, 500, 250, 125, and 62.5 copies/per reaction, respectively).

Completely unmethylated and completely methylated DNA controls were tested with both probes (M-probe and U-probe) separately to identify optimal annealing temperature and temperatures when non-specific binding occurs. Within an annealing temperature range from 50.0°C to 62.0°C, the primer pair revealed clearly distinguishable fluorescence signals up to an annealing temperature of 55°C–56°C for both assays (**Figure 1**). The results of serial dilutions showed a linear correlation between individual dilutions, while R-squared value (R^2^) was 0.9979 and 0.9972 for methylated and unmethylated control, respectively (**Figure 2**). The selectivity for the U-probe was 0.027 (2,7% background), being able to detect 1 unmethylated molecule in the background of 37 methylated. The M-probe with selectivity of 0.003 (0.3% background) is able to detect 1 methylated in the background of 333 unmethylated targets.

### 3.2 Methylation in *GRIA4* is significantly higher in tumor tissue compared to its adjacent non-tumor tissue.

**Figure 3:**
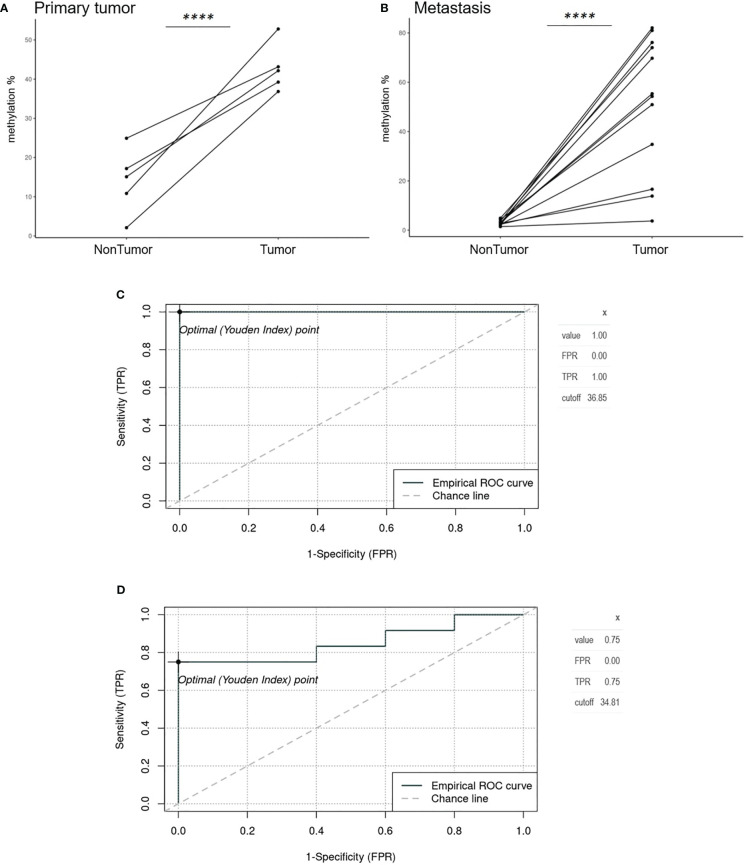
Spaghetti plots comparing tumor and non-tumor tissues of patients with primary tumor and metastasis and ROC analysis. **(A)** Primary tumor tissue versus adjacent non-tumor tissue (colon) (p<0.0001). **(B)** Metastatic tissue and its adjacent non-tumor tissue (liver) (p<0.0001). **(C)** Empirical ROC curve, Youden index, and cutoff for primary tumors (N=5) compared to colon non-tumor tissue (N=5). **(D)** Empirical ROC curve, Youden index, and cut-off for metastases (N=12) compared to colon non-tumor tissue (N=5). ****p ≤ 0.0001.

Average *GRIA4* methylation in primary tumors (N=5) and metastases (N=12) was 42.85% (ranging from 36.85% to 52.81%) and 51.04% (ranging from 3.73% to 81.0%) (**Supplementary Table S3**), respectively, showing no significant difference between these two groups (p = 0.342). Analyzing tissues of patients with primary tumor and patients with metastases separately, primary tumor tissue versus its adjacent non-tumor tissue (colon) showed three times average methylation gain in tumor tissue (**Figure 3A**). On the other hand, an even bigger difference was detected between metastatic tissue and its adjacent non-tumor tissue (liver) (**Figure 3B**), with 17.4 times hypermethylation decrease in liver. ROC analysis defined cutoff values to separate primary tumors (N=5) and metastasis (N=12) from non-tumor colon/rectum (N=5), as the colon/rectum is the tissue where tumors are derived from. Cutoff values were 36.85% for primary tumors (**Figure 3C**) and 34.81% for metastasis (**Figure 3D**). All primary tumors 5/5 (100%) were above this value; for metastases, 9/12 (75%) tissues samples had higher methylation than 34.81%.

### 3.3 Possibility to identify the tissue of origin of metastasis according to the methylation profile

**Figure 4:**
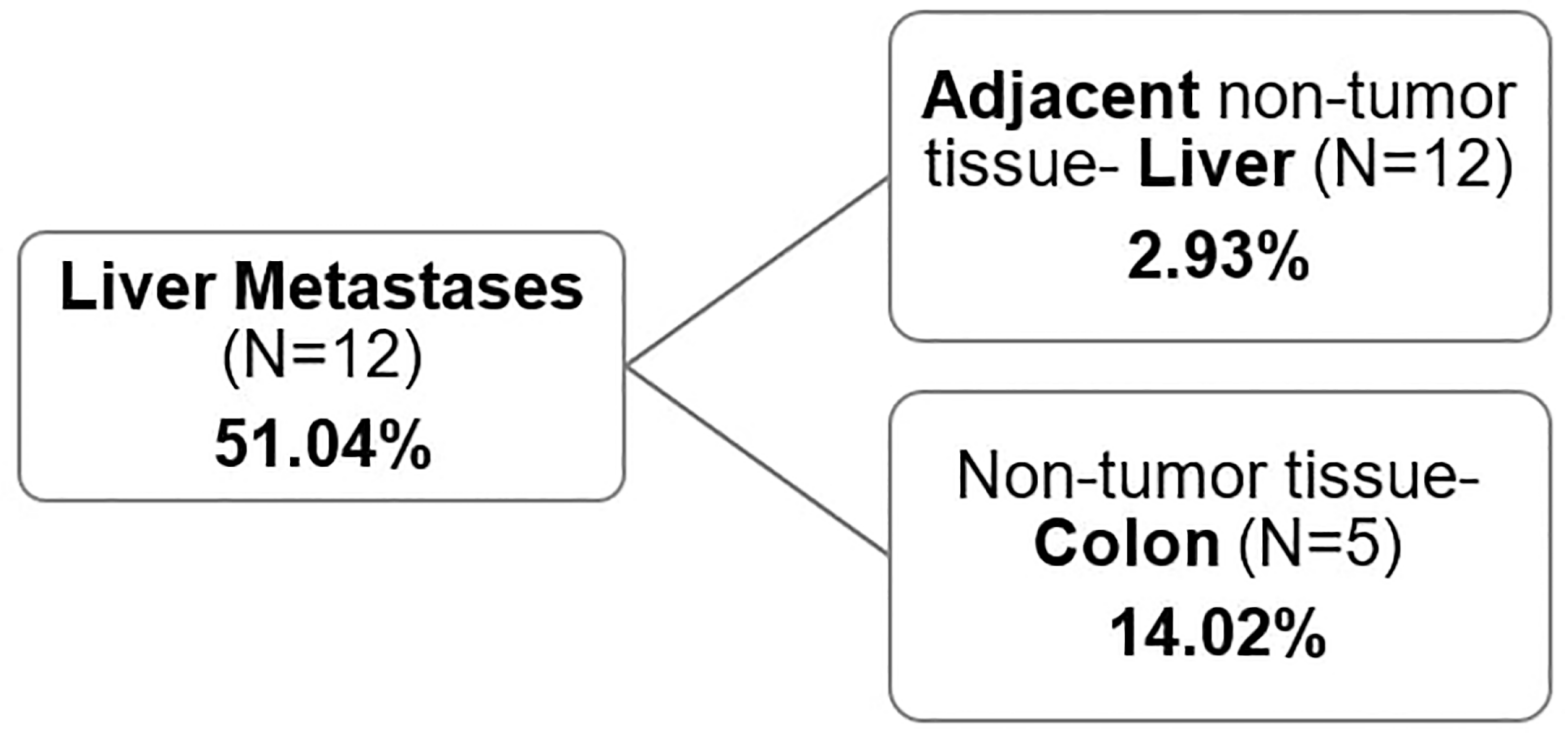
Average *GRIA4* methylation of metastasis and non-tumor tissue. Non-tumor tissue was either the adjacent non-tumor liver (N=12) or non-tumor colon/rectum from different set of patients (N=5).

Non-tumor tissue of primary tumor patients (colon/rectum), with average methylation of 14.02% (ranging from 2.09 to 24.92) (N=5) had almost five times higher *GRIA4* methylation status compared to non-tumor tissue of metastatic patients (liver), ranging from 1.44% to 4.87% (average 2.93%) (**Supplementary Table S3**), p= 0.012. When comparing the methylation status of metastatic tissue *vs*. non-tumor colon/rectum and liver, a smaller increase was observed in liver metastasis in combination with the colon tissue (3.6 times gain) than in liver metastasis in combination with adjacent liver tissue (17.4 times gain) (**Figure 4**). This finding indicates a stronger similarity between liver metastasis and colon/rectum, in contrast to the liver, given that the colon serves as the tissue of origin for the metastasis.

### 3.4 Increased *GRIA4* methylation status in patients’ plasma compared to healthy individuals’ plasma

**Figure 5:**
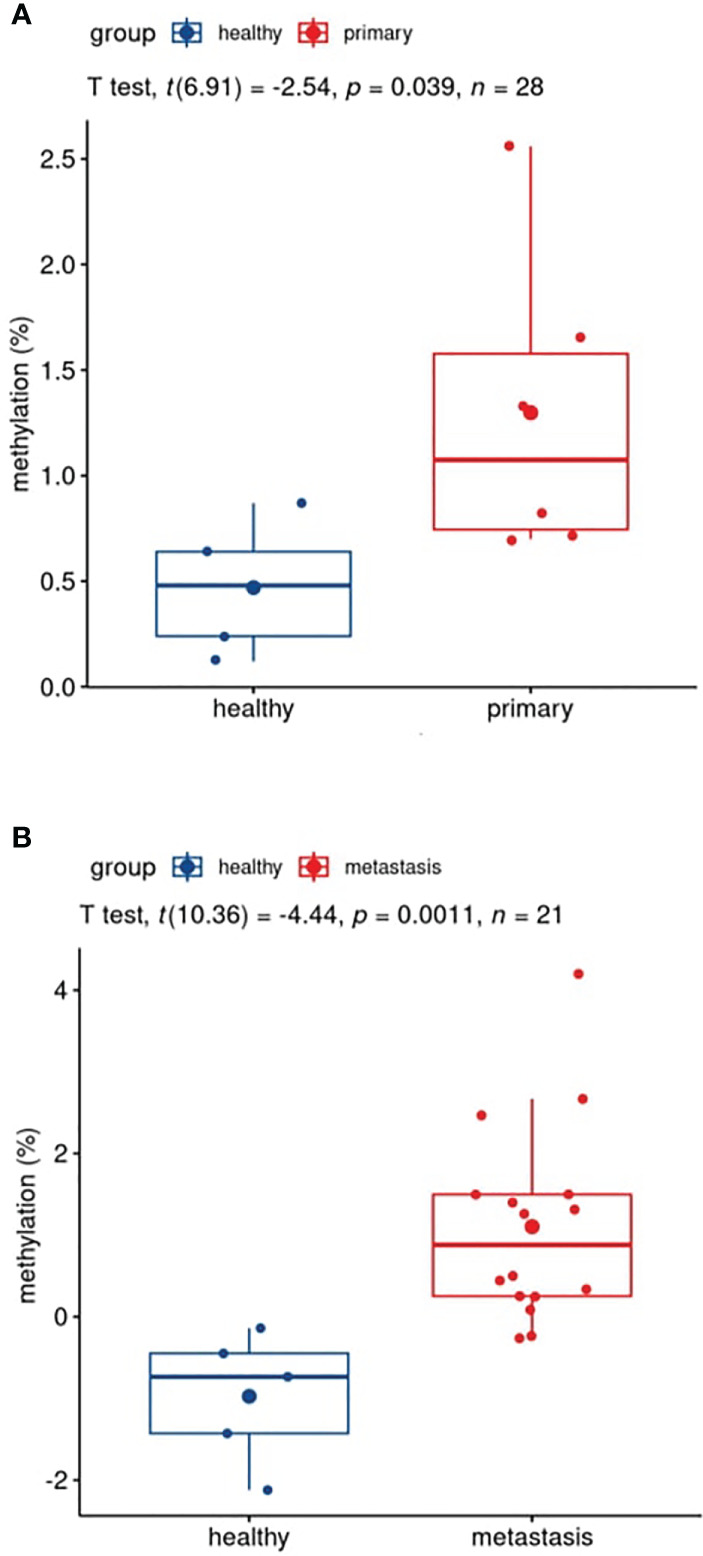
Boxplots of patients’ versus healthy participants’ plasma comparison. **(A)** Pre-surgery plasma of patients with primary tumor *vs*. plasma of healthy individuals; **(B)** pre-surgery plasma of patients with metastasis *vs*. plasma of healthy individuals.

The plasma sample of 22 patients, 6 primary tumor and 16 metastatic patients, taken before surgical removal of tumor was compared with 5 healthy volunteers’ plasma. In primary tumor plasma, methylation percentage ranged between 0.70% and 1.66% (average, 1.30%); in metastatic plasma, it was 7.69% (ranging from 0.77% to 66.75%) and in healthy volunteers (N=5), values were between 0.12% and 0.87% (average, 0.47%) (**Supplementary Table S3**). Both primary (**Figure 5A**) and metastatic plasmas (**Figure 5B**) showed statistically significantly different mean methylation in comparison with healthy plasma, with p=0.039 and p= 0.0011, respectively. *GRIA4* hypermethylation was, on average, 2.8 and 16.4 times higher in primary tumor and metastatic plasma than in healthy individuals.

### 3.5 Significant reduction of *GRIA4* methylation in post-surgery plasma of metastatic patients compared to pre-surgery plasma

**Figure 6:**
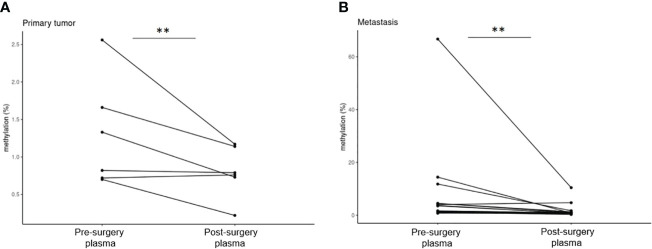
Comparison of pre- and post-surgery plasma of primary tumor and metastatic patients. **(A)** Pre- and post-surgery plasma of primary tumor patients (p=0.037); **(B)** pre- and post-surgery plasma of metastatic patients (p=0.023). **p ≤ 0.01.

Pre-surgery plasma samples showed *GRIA4* methylation ranging from 0.70% to 2.56% (average, 1.30%) in primary tumor patients; in metastatic patients, it ranged from 0.77% to 66.75% (average, 7.69%), and when comparing these two groups, hypermethylation in metastatic plasma was, on average, 5.9 times higher (p=0.051). For post-surgery plasma, average *GRIA4* methylation was 0.80% (ranging from 0.22% to 1.17%) and 1.70% (ranging from 0.28% to 10.44%) for primary and metastatic patients, respectively (**Supplementary Table S3**). When comparing pre- and post-surgery plasma of these two groups separately, there was an average of 1.6 times decrease in post-surgery plasma from primary tumor patients (p=0.037) (**Figure 6A**); on the other hand, metastatic patients’ plasma showed, even bigger, 4.5 times decrease after surgical tumor removal (p=0.023) (**Figure 6B**).

## 4 Discussion

In terms of incidence, colorectal cancer is the third most common cancer worldwide, the second most prevalent in women and the third in men (1, 2). CRC development proceeds through the accumulation of genetic and epigenetic changes. DNA methylation, as the most common epigenetic modification, is an early step of colorectal carcinogenesis (50). In the last few decades, a large number of cancer-related methylated genes have been identified in colorectal carcinoma patients (17–23).

In the current work, we focused on detecting hypermethylation status of gene *GRIA4* in CRC patients using primary tumor/metastatic tissue and complementary pre- and post-surgical plasma samples. We have decided to choose this gene based on the previous studies, where it showed high methylation status and possible detectability from different sample types, such as plasma or stool (27–29). Droplet digital PCR method was chosen based on high sensitivity and our previous experiences with this method and circulating DNA detection (51).

Our results showed significant *GRIA4* hypermethylation increase in primary tumors and liver metastases compared to their adjacent non-tumor tissues (colon, rectum, or liver). Average methylation increase was smaller in patients with primary tumor compared to metastatic patients (3× *vs*. 17.4× gain). Even if the metastasis is localized on the liver, it is not its tissue of origin, so we compared metastasis *vs*. non-tumor colon/rectum, even though the colon/rectum tissues were from other patients (P1_PT–P5_PT). A smaller gain (3.6 times) was observed between metastasis and non-tumor colon compared to metastasis and adjacent liver, indicating a higher degree of similarity between these two sample types. This similarity can be attributed to the fact that the colon is the tissue from which the metastasis originates. Additionally, in the study by Barault et al. (27), it was *GRIA4* that was the most hypermethylated gene in non-tumor colon tissue compared to the other tested biomarkers, which confirms our findings that increased hypermethylation of this gene is present physiologically in the colon/rectum tissue. As methylation is highly tissue specific (52), it can be used to classify tumor subtypes, such as adrenocortical carcinoma (53), hepatocellular carcinoma (54), and cancer of unknown primary sites (55), and it can be used to identify the tissue of origin of metastasis (56). The fact that the cells at the metastatic site have similar methylation patterns suggests that they have retained some of the characteristics of the primary tumor and original tissue. It may be a useful tool to assign original site to metastasis, although additional analysis of primary tumor and metastases from same patient together with methylation status of other tissues and organs have to be performed to provide more precise data.

ROC analysis selected cutoff values to separate primary tumors and metastasis of clinically known colorectal cancer from non-tumor colon tissue. Our data suggest that all primary tumors (5/5) showed above-threshold methylation. These findings are complementary to results from previously published studies where tumors showed *GRIA4* hypermethylation in all 82 tissue samples, ranging from 18% to 97% (27), or in 99.1% (28) of 115 CRC tissues.

Liquid biopsy is a non-invasive way to obtain cancer-derived genetic material for a molecular analysis and monitor relapse or therapy response (12–16). We showed that *GRIA4* hypermethylation from the tumor was detectable in liquid biopsy plasma samples. Plasma of metastatic patients was, on average, 16.4 times higher than that of healthy individuals; in primary tumor patients, a 2.8 times gain was detected. Smaller difference in primary tumor patients can be due to the fact that the primary tumors came from the CRC patients with earlier stages, II (N=5) or III (N=1), and it is known that promoter hypermethylation correlates with tumor stage (57). Moreover, early tumor stages release less circulating DNA into the bloodstream (11, 51). However, in order to enhance future applications, it would be advantageous to conduct a comparative analysis between plasma and stool samples for the *GRIA4* methylation detection of CRC, as stool-based tests utilizing *SEPT9* methylation have demonstrated superior performance compared to plasma-based tests (58, 59). Furthermore, Vega-Benedetti and colleagues (29) proposed *GRIA4* as a potential biomarker for early CRC detection specifically from stool samples. Considering these findings, a comprehensive assessment comparing the diagnostic efficacy of plasma and stool samples would be valuable for advancing the field.

Subsequently, we compared *GRIA4* methylation status in plasma before and after surgical removal of primary tumor/metastasis. In both groups, a decrease in hypermethylation occurred; only three cases showed a slight increase in percentage (average, 0.25%). In the cohort of primary tumor patients, we observed a modest reduction of 1.6-fold in the *GRIA4* methylation status, which may potentially be attributed to the enrollment of individuals in the initial stages of the disease. For metastatic patients, we detected a significant decrease in hypermethylation in post-surgery plasma, by 4.5 times. Although, the current sample size employed in this study is relatively limited, necessitating the acquisition of a more extensive sample cohort to substantiate the role of hypermethylated *GRIA4* as a reliable biomarker for the early non-invasive detection of metastasis of clinically diagnosed cases of colorectal cancer. Additionally, it is important to note that there was substantial variability in methylation levels across the samples; therefore, it may be advantageous to explore the integration of complementary biomarkers in conjunction with *GRIA4*.

Significant *GRIA4* methylation decrease in post-surgery plasma indicates that this biomarker holds promise as a robust candidate for simple and cost-effective CRC detection using ddPCR, a common platform in oncology labs. Although it would be beneficial to incorporate additional biomarkers, as already mentioned above, our primary objective was to identify a specific biomarker that can contribute to the development of straightforward, single-gene tests like Epi proColonTM (24) or ColoSureTM (25). Further studies on already identified biomarkers could facilitate their progressive implementation into clinical diagnostics, as seen with the *SEPT9* or *Vimentin* genes (60–63).

## 5 Conclusion

This methylation-specific ddPCR assay proved to be a suitable detection method for capturing the hypermethylated *GRIA4* gene from conventional tissue as well as liquid biopsy samples. Our data suggest, that this biomarker could serve as a tool to identify colorectal cancer and its metastasis from both tissue and plasma samples, furthermore, it may aid in determining the specific tissue of origin for the metastatic lesions. We observed a significant increase in *GRIA4* methylation in the plasma of metastatic patients, with a remarkable 16.4-fold amplification, which nominates this gene as potential novel biomarker for an early non-invasive detection of metastasis of clinically known CRC, however, additional analysis of a larger sample cohort must be performed.

## Data availability statement

The authors acknowledge that the data presented in this study must be deposited and made publicly available in an acceptable repository, prior to publication. Frontiers cannot accept a article that does not adhere to our open data policies.The datasets presented in this study are available on https://data.mendeley.com/datasets/sm5n4bgtf3.

## Ethics statement

The studies involving human participants were reviewed and approved by Ethics committee of the Jessenius Faculty of Medicine in Martin, Comenius University in Bratislava (number of approval 1863/2016). The patients/participants provided their written informed consent to participate in this study.

## Author contributions

EL, TB, PP was responsible for experimental design, data analysis and wrote the manuscript, EL, TB, ET, IK, AV performed the experiments, ZL secured financial funding, was responsible for experimental design, supervised the data analysis and interpretation, and critically reviewed and revised the manuscript drafts, MG performed bioinformatic analyses, generated the figures and revised the manuscript drafts, LL, PM, EK, JM performed the histopathological evaluation and collected patient´s samples. All authors contributed to the article and approved the submitted version.
