# Supplementary material for: Hypermethylated GRIA4, a potential biomarker for an early non-invasive detection of metastasis of clinically known colorectal cancer
Source: Front Oncol. 2023 Jul 5;13:1205791. doi: 10.3389/fonc.2023.1205791 (PMC10354553; doi:10.3389/fonc.2023.1205791)
Supplement: Supplementary file 1 [file Table_1.docx]

| Patient number | Primary (P) or Metastatic (M) patient | Tumor tissue | Non-tumor tissue | Plasma before surgery | The first postoperative blood sample (2-5 days) |
| --- | --- | --- | --- | --- | --- |
| P1_PT | **P** | 🗸 | 🗸 |  |  |
| P2_PT | **P** | 🗸 | 🗸 | 🗸 | 🗸 |
| P3_PT | **P** | 🗸 | 🗸 | 🗸 | 🗸 |
| P4_PT | **P** | 🗸 | 🗸 | 🗸 | 🗸 |
| P5_PT | **P** | 🗸 | 🗸 | 🗸 | 🗸 |
| P6_MTS | **M** | 🗸 | 🗸 | 🗸 | 🗸 |
| P7_MTS | **M** | 🗸 | 🗸 | 🗸 | 🗸 |
| P8_MTS | **M** | 🗸 | 🗸 | 🗸 | 🗸 |
| P9_MTS | **M** | 🗸 | 🗸 | 🗸 | 🗸 |
| P10_MTS | **M** | 🗸 | 🗸 | 🗸 | 🗸 |
| P11_MTS | **M** | 🗸 | 🗸 | 🗸 | 🗸 |
| P12_MTS | **M** | 🗸 | 🗸 | 🗸 | 🗸 |
| P13_MTS | **M** | 🗸 | 🗸 | 🗸 | 🗸 |
| P14_MTS | **M** | 🗸 | 🗸 | 🗸 | 🗸 |
| P15_MTS | **M** | 🗸 | 🗸 | 🗸 | 🗸 |
| P16_MTS | **M** | 🗸 | 🗸 | 🗸 | 🗸 |
| P17_MTS | **M** | 🗸 | 🗸 | 🗸 | 🗸 |
| P18_PT | **P** |  |  | 🗸 | 🗸 |
| P19_PT | **P** |  |  | 🗸 | 🗸 |
| P20_MTS | **M** |  |  | 🗸 | 🗸 |
| P21_MTS | **M** |  |  | 🗸 | 🗸 |
| P22_MTS | **M** |  |  | 🗸 | 🗸 |
| P23_MTS | **M** |  |  | 🗸 | 🗸 |

**Supplementary Table 1.** **Information about 23 patients included in this study.** 7 patients with primary tumor and 16 patients with liver metastasis of clinically known colorectal cancer. We obtained tumor tissues of 17 patients (5 primary and 12 metastatic) and blood samples of 22 patients (6 primary and 16 metastatic).
