# Supplementary material for: Hypermethylated GRIA4, a potential biomarker for an early non-invasive detection of metastasis of clinically known colorectal cancer
Source: Front Oncol. 2023 Jul 5;13:1205791. doi: 10.3389/fonc.2023.1205791 (PMC10354553; doi:10.3389/fonc.2023.1205791)
Supplement: Supplementary file 2 [file Table_2.docx]

| Primary tumor patient ID | Stage | MKCH-10 |
| --- | --- | --- |
| P1_PT | III | C18.4 |
| P2_PT | II | C18.7 |
| P3_PT | II | C18.2 |
| P4_PT | II | C18.2 |
| P5_PT | II | C19 |
| P18_PT | III | C20 |
| P19_PT | II | C20 |

**Supplementary Table 2. Pathological information about primary tumor patients.** Seven primary tumor patients were included in this study. Five of them with stage II and two with stage III CRC.
