# Supplementary material for: Hypermethylated GRIA4, a potential biomarker for an early non-invasive detection of metastasis of clinically known colorectal cancer
Source: Front Oncol. 2023 Jul 5;13:1205791. doi: 10.3389/fonc.2023.1205791 (PMC10354553; doi:10.3389/fonc.2023.1205791)
Supplement: Supplementary file 3 [file Table_3.docx]

| Patient number | Tumor tissue | Non-tumor tissue | Plasma before surgery | The first postoperative blood sample (2-5 days) |
| --- | --- | --- | --- | --- |
| P1_PT | 39,25% | 17,18% |  |  |
| P2_PT | 52,81% | 10,84% | 0,72% | 0,76% |
| P3_PT | 82,05% | 3,56% | 66,75% | 10,44% |
| P4_PT | 36,85% | 2,09% | 1,66% | 1,14% |
| P5_PT | 42,16% | 15,09% | 1,33% | 0,73% |
| P6_MTS | 50,92% | 4,37% | 3,72% | 0,71% |
| P7_MTS | 69,74% | 2,36% | 14,43% | 0,79% |
| P8_MTS | 81,00% | 2,06% | 3,53% | 0,82% |
| P9_MTS | 43,16% | 24,92% | 0,70% | 0,22% |
| P10_MTS | 54,26% | 3,11% | 4,05% | 4,74% |
| P11_MTS | 55,35% | 3,14% | 1,29% | 0,60% |
| P12_MTS | 3,73% | 1,44% | 1,09% | 0,28% |
| P13_MTS | 34,81% | 2,31% | 4,48% | 1,29% |
| P14_MTS | 13,83% | 2,79% | 0,77% | 0,81% |
| P15_MTS | 16,61% | 2,35% | 1,56% | 0,62% |
| P16_MTS | 74,03% | 4,87% | 4,47% | 0,98% |
| P17_MTS | 76,13% | 2,81% | 11,81% | 1,69% |
| P18_PT |  |  | 2,56% | 1,17% |
| P19_PT |  |  | 0,82% | 0,79% |
| P20_MTS |  |  | 1,40% | 0,79% |
| P21_MTS |  |  | 1,65% | 0,98% |
| P22_MTS |  |  | 1,28% | 0,97% |
| P23_MTS |  |  | 0,79% | 0,61% |

**Supplementary Table 3: Percentage values of *GRIA4* methylation in tissue and plasma samples.** The quantity of methylation was expressed in percentage as the ratio of methylated sequences to the sum of methylated and unmethylated sequences**.**
